# Effects of phosphodiesterase V inhibition alone and in combination with BNP on cardiovascular and renal response to volume load in human preclinical diastolic dysfunction

**DOI:** 10.14814/phy2.14974

**Published:** 2021-08-17

**Authors:** Siu‐Hin Wan, Paul M. McKie, Joshua P. Slusser, John C. Burnett, David O. Hodge, Horng H. Chen

**Affiliations:** ^1^ Division of Cardiology Department of Internal Medicine University of Texas—Southwestern Medical Center Dallas TX USA; ^2^ Department of Cardiovascular Diseases Cardiorenal Research Laboratory Mayo Clinic and Foundation Rochester MN USA; ^3^ Department of Health Sciences Research Mayo Clinic and Foundation Rochester MN USA

## Abstract

Preclinical diastolic dysfunction (PDD) results in impaired cardiorenal response to volume load (VL) which may contribute to the progression to clinical heart failure with preserved ejection fraction (HFpEF). The objective was to evaluate if phosphodiesterase V inhibition (PDEVI) alone or combination PDEVI plus B‐type natriuretic peptide (BNP) administration will correct the impaired cardiorenal response to VL in PDD. A randomized double‐blinded placebo‐controlled cross‐over study was conducted in 20 subjects with PDD, defined as left ventricular ejection fraction (LVEF) >50% with moderate or severe diastolic dysfunction by Doppler echocardiography and without HF diagnosis or symptoms. Effects of PDEVI with oral tadalafil alone and tadalafil plus subcutaneous (SC) BNP, administered prior to acute volume loading, were assessed. Tadalafil alone did not result in improvement in cardiac response to VL, as measured by LVEF, LV end diastolic volume, left atrial volume (LAV), or right ventricular systolic pressure (RVSP). Tadalafil plus SC BNP resulted in improved cardiac response to VL, with increased LVEF (4.1 vs. 1.8%, *p* = 0.08) and heart rate (4.3 vs. 1.6 bpm, *p* = 0.08), and reductions in both LAV (−4.3 ± 10.4 vs. 2.8 ± 6.6 ml, *p* = 0.03) and RVSP (−4.0 ± 3.0 vs. 2.1 ± 6.0 mmHg, *p* < 0.01) versus tadalafil alone. Plasma and urinary cyclic guanosine monophosphate (cGMP) excretion levels were higher (11.3 ± 12.3 vs. 1.7 ± 3.8 pmol/ml, 1851.0 ± 1386.4 vs. 173.4 ± 517.9 pmol/min, *p* < 0.01) with tadalafil plus SC BNP versus tadalafil alone. There was no improvement in renal response as measured by GFR, renal plasma flow, sodium excretion, and urine flow with tadalafil plus SC BNP compared to tadalafil alone. In subjects with PDD, tadalafil alone resulted in no improvement in cardiac adaptation, while tadalafil and SC BNP resulted in enhanced cardiac adaptation to VL.

**Trial Registration:**

ClinicalTrials.gov NCT01544998.

## 1 INTRODUCTION

There are over 1 million heart failure (HF) hospitalizations and over $30 billion in heart failure‐related costs in the United States annually, and approximately half of patients with HF have preserved ejection fraction (HFpEF) (Benjamin et al., 2018). The precursor to HFpEF is preclinical diastolic dysfunction (PDD), defined as left ventricular diastolic dysfunction with normal systolic function and without heart failure signs or symptoms (Wan et al., 2014). PDD resides in the American College of Cardiology/American Heart Association (ACC/AHA) Stage B HF (Yancy et al., 2013). PDD is common in the general population, and those with PDD are at risk of developing symptomatic HFpEF (Lam et al., 2011; Redfield et al., 2003).

In patients with PDD, the cyclic guanosine monophosphate (cGMP) pathway is dysfunctional when responding to a volume load (VL) resulting in impaired cardiorenal adaptation as compared to healthy controls (McKie et al., 2011). The natriuretic peptide (NP) system plays an important role in the modulation of myocardial, renal, and vascular function in HF through the second messenger cyclic 3’‐5’‐guanosine monophosphate (cGMP). Cyclic GMP has been shown to potentiate cardiorenal adaptation to VL in animal models, and it can be increased through inhibition of phosphodiesterase V (PDEV) degradation or exogenous administration of BNP (Chen et al., 2006; Forfia et al., 2007).

Given that PDEV degrades cGMP and results in cGMP attenuation, this study seeks to assess the cardiorenal effects of PDEV inhibition on VL in PDD. We have previously demonstrated that administration of exogenous B type natriuretic peptide (BNP) leading to cGMP activation results in restoration of cardiorenal adaptation to VL and improvement in diastolic dysfunction among humans with PDD (Wan et al., 2016). Therefore, we further wish to assess the effects of PDEV inhibition (PDEVI) alone and in combination with BNP administration.

As there are currently no known approved therapies for HFpEF or PDD, the cardiorenal effects of combination therapies are important to elucidate as there may be synergistic effects beyond individual therapy. While individual therapeutic strategies have been studied, the cardiorenal effects of combination PDEV inhibition and exogenous BNP administration are unknown in humans with PDD.

The objective of this study is to define the cardiac and renal adaptations to VL with PDEVI alone (tadalafil) and combination of tadalafil plus BNP in patients with PDD. We hypothesize that the combination therapy of tadalafil plus BNP will result in a greater improvement in cardiorenal adaptation to VL as compared to tadalafil alone.

## 2 METHODS

### 2.1 Study design

The study is a double‐blinded, placebo‐controlled, cross‐over study to compare tadalafil alone versus tadalafil plus subcutaneous (SC) BNP on cardiorenal responses to saline VL in subjects with PDD. This study was approved by the Mayo Foundation Institutional Review Board and was performed at the Clinical Research Unit at Saint Mary's Hospital, Mayo Clinic Rochester Minnesota. Written informed consent was obtained from all participants.

### 2.2 Study population

**FIGURE 1:**
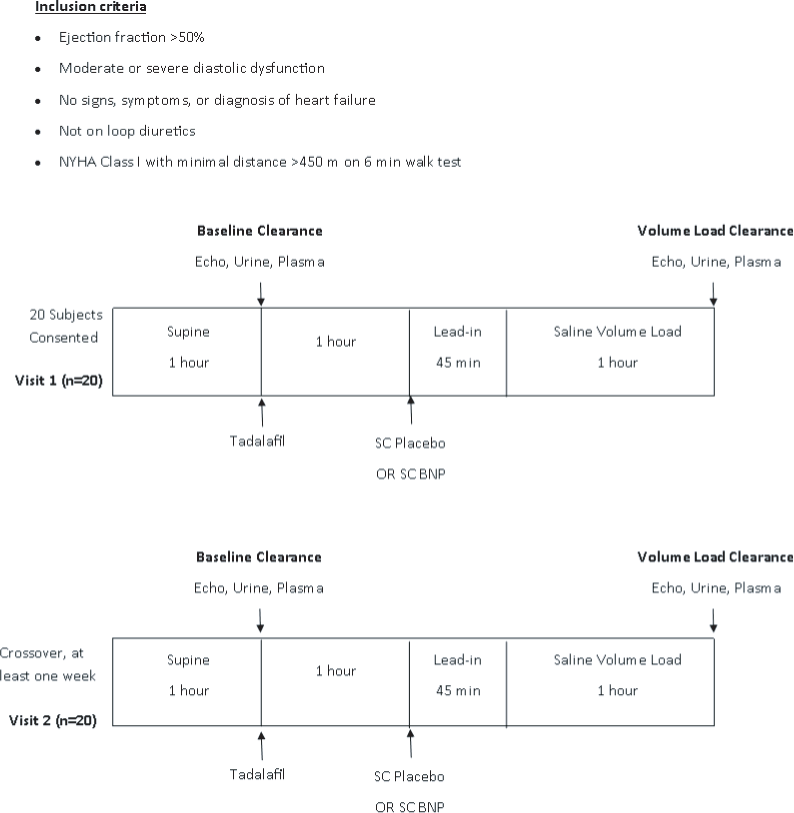
Study design. BNP, B type natriuretic peptide; Echo, echocardiogram; SC, subcutaneous

Twenty patients with PDD (AHA Stage B HF) were enrolled (Figure 1) and randomized to receive oral tadalafil and SC placebo or oral tadalafil and SC BNP before VL. Cardiac and renal assessments, transthoracic echocardiography, urine, and plasma analyses were performed at baseline and 60 min after VL. All patients returned for a second visit and underwent the cross‐over arm of the study.

### 2.3 Inclusion criteria

Inclusion criteria include the following: ejection fraction greater than 50%; moderate or severe diastolic dysfunction as assessed by Doppler echocardiography; no current or previous diagnosis of HF; not on loop diuretics; and New York Heart Association Class 1 with minimal distance >450 meters on 6‐minute walk test in the absence of mechanical limitations (Wan et al., 2014). Cardiovascular medications were at stable doses for at least 2 weeks prior to study entry.

### 2.4 Echocardiographic assessment

Based on the American Society of Echocardiography recommendations, standard acoustic window echocardiographic images were obtained (Lang et al., 2015). Biplane Simpsons method of disks was used to determine ventricular volumes and 2D ejection fraction was obtained. Mitral inflow pulsed wave Doppler examination and tissue Doppler imaging of the mitral annulus were used to determine left ventricular (LV) diastolic function and filling pressures. All echocardiographic data were obtained by a certified sonographer and interpreted by HHC who was blinded to the assigned treatment.

### 2.5 Study protocol

Subjects received instructions about a controlled salt diet (120 mEq Na/day) for 1 week prior to study initiation and were compliant by self‐reporting. Twenty‐four‐hour urine collection was obtained 1 day prior to the active renal clearance study day for assessment of sodium excretion, creatinine clearance, and microalbuminuria. If the 24‐h urine collection revealed that subject has been non‐compliant with diet, diet instructions were given again and the subject returned after at least 1 week with repeat 24‐h urine collection. Hematology and biochemistry labs, a 6‐min walking test, and a physical examination with vital signs were obtained. One day prior to the study date, subjects who met inclusion criteria were recruited and admitted to the Clinic Research Unit (CRU) at St. Mary's Hospital, Mayo Clinic CTSA, Rochester, Minnesota (Figure 1).

Subjects received regular medications on their study day, except for anti‐glycemic agents. These were postponed until after the last renal clearance measurement. To ensure good urine flow, subjects were hydrated orally with 10 ml/kg of water.

Subjects were then placed in supine position for 1 h. Two standard intravenous catheters were placed, one in each arm, for infusion and blood sampling. Intravenous iothalamate and para‐amino‐hippurate (PAH) were administered. Venous blood samples and renal clearances were obtained at 60 min. Measurements of the urine and blood were made: urinary flow (ml); urinary sodium excretion (UNaV) (mEq/min); urinary cGMP excretion (UcGMP) (pmol/min); and blood sodium mEQ/L and cGMP pmol/ml. Continuous monitoring by ECG and blood pressures were obtained in subjects. An echocardiogram was obtained for left atrial (LA) and LV volumes and systolic and diastolic functions.

Subjects were then randomized to receive oral tadalafil 5 mg and SC placebo or oral tadalafil 5 mg and SC BNP 10 µg/kg (Scios, Mountain View, California). Based on prior studies and to minimize hypotension, a tadalafil dose of 5 mg was used (Wan et al., 2019). Tadalafil was administered orally and after 1 h, SC placebo or BNP was given in the abdominal subcutaneous tissue. After a 45‐min lead‐in period, an acute saline load was administered (0.9% NaCl at 0.25 ml/kg/min for 1 h). Venous blood and renal samples were obtained at 60 min. Immediately after the end of the acute VL, an echocardiogram was performed. Subjects returned in 1 week or later for the cross‐over portion of the study.

### 2.6 Neurohormonal, electrolyte, and renal assessment

Plasma atrial natriuretic peptide (ANP), BNP, aldosterone, angiotensin II, and urine cGMP were obtained and determined by radioimmunoassay as previously described (McKie et al., 2011). Plasma and urine concentration of iothalamate and PAH as well as creatinine were measured by the Mayo Core Renal lab.

### 2.7 Statistical methods

Continuous variables are presented as mean ± SD and discrete variables as frequency (percentage). Comparisons between the two treatment groups (tadalafil and SC placebo, and tadalafil and SC BNP) were made using the Student's *t*‐test for normally distributed continuous variables, the rank‐sum test for continuous variables with a skewed distribution, and the Pearson Chi‐squared test for independence for categorical variables. Comparisons within groups (between visit 1 and visit 2) were made using a paired *t*‐test. For all analyses, statistical significance was accepted as *p* < 0.05. Statistical analyses were completed with SAS 9.4 (SAS Institute Inc, Cary, North Carolina).

## 3 RESULTS

The baseline characteristics of the study population are shown in Table 1. The average age was 71.2 years with 40% females. The average heart rate was 61 bpm and the average blood pressure was 130/73 mmHg. The population tended to be obese (mean BMI 33 kg/m^2^). Comorbidities such as diabetes mellitus (25%), coronary artery disease (45%), and history of hypertension (75%) were common. The average left ventricular ejection fraction (LVEF) was 58.4%, left ventricular end diastolic volume (LVEDV) was 152.6 ml, right ventricular systolic pressure (RVSP, as estimated by the modified Bernoulli equation P = 4v^2^ with tricuspid regurgitant jet peak velocity) was 31.8 mmHg, E/e’ was 14.3, and left atrial volume (LAV) was 80.8 ml. The average ANP and BNP values were low, 43.5 pg/ml and 79.9 pg/ml, respectively. Similarly, both mean plasma aldosterone and angiotensin II levels were within the normal range. Despite subjects having diastolic dysfunction and LA enlargement, both ANP and BNP levels are low with no activation of the angiotensin aldosterone system, consistent with asymptomatic diastolic dysfunction. The characteristics of the study population are thus similar to that previously reported (McKie et al., 2011).

**TABLE 1:** Baseline characteristics of the study population

Variable	Subjects (*n* = 20)
Age (years), mean ± SD	71.2 ± 8.8
Female, *n* (%)	8 (40)
Heart rate (beats per min), mean ± SD	60.9 ± 8.3
Blood pressure (mmHg), mean ± SD	
Systolic	130 ± 16.1
Diastolic	72.6 ± 10.4
Body weight (kg), mean ± SD	97.4 ± 16.8
Body mass index (BMI, kg/m^2^), mean ± SD	33 ± 5.3
GFR (ml/min), mean ± SD	62 ± 16.4
Diabetes mellitus, *n* (%)	5 (25)
Coronary artery disease, *n* (%)	9 (45)
Myocardial infarction, *n* (%)	4 (20)
History of Hypertension, *n* (%)	15 (75)
ACEI or ARB, *n* (%)	12 (60)
Beta blocker, *n* (%)	14 (70)
Thiazide diuretics, *n* (%)	6 (30)
LV ejection fraction (%), mean ± SD	58.4 ± 9.3
LV end systolic volume (ml), mean ± SD	64.1 ± 30.1
LV end diastolic volume (ml), mean ± SD	152.6 ± 42.4
LV end systolic diameter (cm), mean ± SD	3.4 ± 0.6
LV end diastolic diameter (cm), mean ± SD	5.3 ± 0.5
RV systolic pressure (mmHg), mean ± SD	31.8 ± 4.9
LAV (ml), mean ± SD	80.8 ± 20.6
E/e’ (medial), mean ± SD	14.3 ± 3.9
ANP (pg/ml), mean ± SD	43.5 ± 41.6
BNP (pg/ml), mean ± SD	79.9 ± 45.5
Aldosterone (ng/dl), mean ± SD	6.8 ± 9.9
Angiotensin II (pg/ml), mean ± SD	2.0 ± 1.3
Diastolic grade 2, *n* (%)	20 (100)

Abbreviations: ACEI, angiotensin‐converting enzyme inhibitor; ANP, atrial natriuretic peptide; ARB, angiotensin receptor blocker; BNP, B‐type natriuretic peptide; BNP, brain natriuretic peptide; cGMP, cyclic guanosine monophosphate; E/e’, E velocity/e’ velocity; LA, left atrium; LV, left ventricle; LVEDV, left ventricle end diastolic volume; LVESV, left ventricle end systolic volume; LVEDD, left ventricle end diastolic diameter; LVESD, left ventricle end systolic diameter; LAV, left atrial volume; RVSP, right ventricle systolic pressure.

### 3.1 Cardiac adaptation to volume load

With tadalafil alone, there was a decrease in systolic and diastolic blood pressures and trend for an increase in heart rate with intravenous saline VL (SBP 137.5 vs. 130.2 mmHg, *p* < 0.01; DBP 68.3 vs. 63.2 mmHg, *p* = 0.05; HR 57.7 vs. 59.3 bpm, *p* = 0.07) (Table 2). There was no change in ventricular adaptation or function with VL as demonstrated by LVEF (59.8 vs. 62.4%, *p* = 0.23), LVEDV (155.1 vs. 151.6 ml, *p* = 0.90), RVSP (31.9 vs. 33.6 mmHg, *p* = 0.20), or LAV (82.5 vs. 87.4 ml, *p* = 0.10).

**TABLE 2:** Tadalafil and SC placebo versus tadalafil and SC BNP at baseline and after normal saline volume load (VL)

Variable	Tadalafil and SC Placebo			Tadalafil and SC BNP		
	Baseline (*n* = 20)	Volume load (*n* = 20)	*p* value (Baseline vs. VL)	Baseline ( *n* = 20)	Volume load ( *n* = 20)	*p* value (Baseline vs. VL)
Systolic BP (mmHg)	137.5 ± 15.2	130.2 ± 17.3	<0.01	136.5 ± 15.7	124.1 ± 11.6	<0.01
Diastolic BP (mmHg)	68.3 ± 13.0	63.2 ± 13.0	0.05	68.4 ± 15.2	63.0 ± 11.0	0.09
Heart rate (bpm)	57.7 ± 7.3	59.3 ± 9.0	0.07	58.4 ± 9.3	62.6 ± 7.6	0.02
LVEF (%)	59.8 ± 8.5	62.4 ± 7.2	0.23	60.2 ± 8.4	64.6 ± 7.5	<0.01
LV end diastolic volume (ml)	155.1 ± 38.9	151.6 ± 37.9	0.90	153.2 ± 43.6	145.9 ± 41.5	0.05
LV end systolic volume (ml)	66.6 ± 28.1	61.5 ± 28.2	0.34	60.9 ± 28.8	54.9 ± 26.3	<0.01
Cardiac output (L/min)	5.6 ± 1.4	5.5 ± 1.6	0.64	5.2 ± 1.3	5.4 ± 1.2	0.22
E/e’	15.2 ± 4.3	15.3 ± 4.4	0.92	14.9 ± 3.7	14.2 ± 3.6	0.35
RVSP (mmHg)	31.9 ± 4.6	33.6 ± 6.2	0.20	30.7 ± 4.8	27.8 ± 6.4	<0.01
LAV (ml)	82.5 ± 19.6	87.4 ± 22.4	0.10	81.8 ± 20.4	78.3 ± 21.4	0.11
ANP (pg/ml)	39.7 ± 26.4	42.9 ± 40.7	0.69	43.5 ± 41.6	31.7 ± 24.0	0.06
BNP (pg/ml)	62.1 ± 37.9	68.0 ± 42.1	<0.01	79.9 ± 45.5	603.7 ± 656.3	<0.01
cGMP (pmol/ml)	4.1 ± 2.6	5.8 ± 4.8	0.06	4.4 ± 4.3	15.7 ± 13.1	<0.01
Aldosterone (ng/dl)	7.6 ± 13.1	3.9 ± 4.0	0.08	6.8 ± 9.9	3.4 ± 2.4	0.08
Angiotensin II (pg/ml)	2.8 ± 1.8	2.7 ± 2.9	0.90	2.0 ± 1.3	2.1 ± 1.6	0.89
GFR (ml/min)	72.5 ± 33.7	76.4 ± 26.3	0.49	75.8 ± 23.4	68.3 ± 25.9	0.39
Renal plasma flow (ml/min)	298.8 ± 126.1	312.2 ± 96.8	0.57	316.6 ± 99.2	279.5 ± 117.5	0.29
Urine flow (ml/min)	5.5 ± 3.6	7.7 ± 3.0	0.02	5.5 ± 3.0	6.9 ± 3.4	0.11
Sodium excretion (mEq/min)	184.2 ± 94.6	281.2 ± 159.4	0.03	208.9 ± 144.9	301.4 ± 164.0	0.04
Urinary cGMP excretion (pmol/min)	719.8 ± 417.0	893.3 ± 709.0	0.15	735.2 ± 427.6	2586.2 ± 1370.4	<0.01

Values are shown as mean ± SD.

Abbreviations: ANP, atrial natriuretic peptide; BNP, brain natriuretic peptide; BP, blood pressure; cGMP, cyclic guanosine monophosphate; GFR, glomerular filtration rate; LAV, left atrial volume; LVEF, left ventricular ejection fraction; RVSP, right ventricular systolic pressure.

Tadalafil plus SC BNP combination, there was also a decrease in systolic and diastolic blood pressures and increase in heart rate with VL (SBP 136.5 vs. 124.1 mmHg, *p* < 0.01; DBP 68.4 vs. 63.0 mmHg, *p* = 0.09; HR 58.4 vs. 62.6 bpm, *p* = 0.02) (Table 2). However, with tadalafil plus SC BNP, there was an increase in LVEF (60.2 vs. 64.6%, *p* < 0.01), a decrease in LVEDV (153.2 vs. 145.9 ml, *p* = 0.05), a decrease in LV end systolic volume (LVESV) (60.9 vs. 54.9 ml, *p* < 0.01), a decrease in RVSP (30.7 vs. 27.8 mmHg, *p* < 0.01), and a trend for decrease in LAV (81.8 vs. 78.3 ml, *p* = 0.11) in response to VL.

**FIGURE 2:**
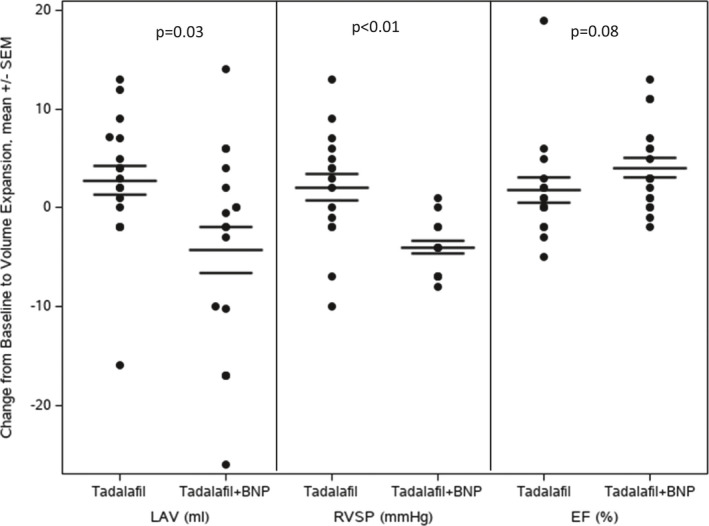
Echocardiographic Parameters and Response to VL in tadalafil and SC placebo versus tadalafil and SC BNP. SC BNP: received tadalafil plus SC BNP. Placebo: received tadalafil plus placebo. EF, ejection fraction; LAV, left atrial volume; RVSP, right ventricular systolic pressure

Tadalafil plus SC BNP as compared to tadalafil alone in response to VL resulted in decrease in LAV (−4.3 vs. 2.8 ml, *p* = 0.03) and RVSP (−4.0 vs. 2.1 mmHg, *p* < 0.01) (Figure 2). There was a trend for greater increase in LVEF in the tadalafil plus SC BNP group (4.1 vs. 1.8%, *p* = 0.08) and heart rate (4.3 vs. 1.6 bpm, *p* = 0.08) (Figure 2).

### 3.2 Renal response to volume load

With tadalafil alone, there was an increase in urine flow (5.5 vs. 7.7 ml/min, *p* = 0.02) and sodium excretion (184.2 vs. 281.2 mEq/min, *p* = 0.03); however, there was no significant increase in GFR, renal plasma flow, or urinary cGMP excretion in response to VL (Table 2).

With tadalafil plus SC BNP, there was an increase in sodium excretion (208.9 vs. 301.4 mEq/min, *p* = 0.04) and urinary cGMP excretion (735.2 vs. 2586.2 pmol/min, *p* < 0.01), but no significant increase in urine flow, GFR, or renal plasma flow in response to VL (Table 2).

**FIGURE 3:**
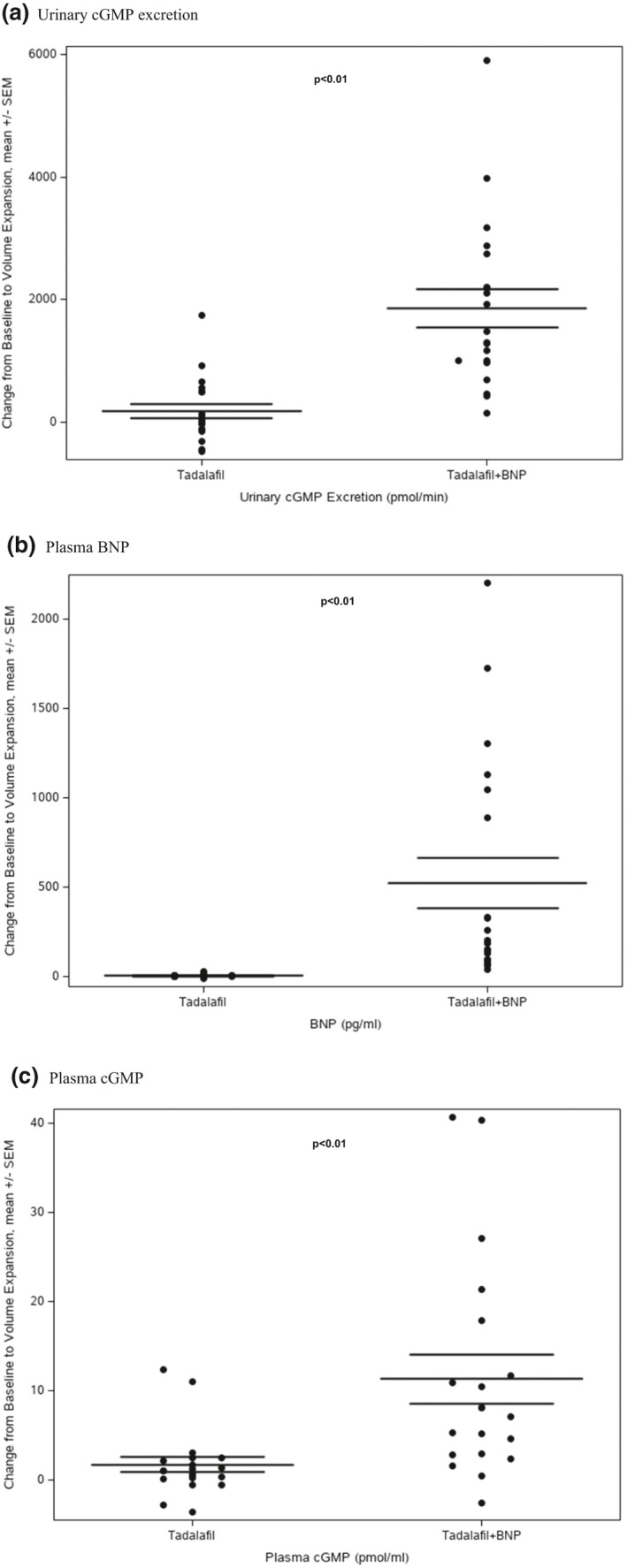
Neurohumoral Parameters and Response to VL in tadalafil and SC placebo versus tadalafil and SC BNP. (a) Urinary cGMP excretion. SC BNP: received tadalafil plus SC BNP. Placebo: received tadalafil plus placebo. (b) Plasma BNP. SC BNP: received tadalafil plus SC BNP. Placebo: received tadalafil plus placebo. (c) Plasma cGMP. SC BNP: received tadalafil plus SC BNP. Placebo: received tadalafil plus placebo. cGMP, cyclic guanosine monophosphate

Comparing tadalafil alone and in combination with tadalafil plus SC BNP, there was no difference in renal response to VL as measured by GFR, renal plasma flow, urine flow, and sodium excretion (Table 3). However, there was an increase in urinary cGMP excretion response with combination tadalafil plus SC BNP compared to tadalafil alone (1851.0 vs. 173.4 pmol/min, *p* < 0.01) (Figure 3a).

**TABLE 3:** Change after normal saline volume load (VL) in tadalafil and SC placebo versus tadalafil and SC BNP

Variable	Tadalafil + Placebo (*n* = 20)	Tadalafil + SC BNP (*n* = 20)	*p* value
Systolic BP (mmHg)	−7.3 ± 10.6	−12.4 ± 14.5	0.21
Diastolic BP (mmHg)	−5.1 ± 10.6	−5.4 ± 13.7	0.97
Heart rate (bpm)	1.6 ± 3.7	4.3 ± 7.3	0.08
LVEF (%)	1.8 ± 5.6	4.1 ± 4.4	0.08
LV End Diastolic volume (ml)	0.5 ± 16.8	−8.1 ± 15.8	0.14
LV End Systolic volume (ml)	−2.3 ± 9.2	−6.9 ± 9.1	0.16
Cardiac output (L/min)	−0.1 ± 0.8	0.2 ± 0.7	0.25
E/e’	0.1 ± 4.6	−0.7 ± 3.3	0.80
RVSP (mmHg)	2.1 ± 6.0	−4.0 ± 3.0	<0.01
LAV (ml)	2.8 ± 6.6	−4.3 ± 10.4	0.03
ANP (pg/ml)	3.2 ± 33.5	−11.8 ± 24.4	0.34
BNP (pg/ml)	5.9 ± 8.5	523.8 ± 634.7	<0.01
cGMP (pmol/ml)	1.7 ± 3.8	11.3 ± 12.3	<0.01
Aldosterone (ng/dl)	−3.7 ± 9.1	−3.4 ± 8.2	0.50
Angiotensin II (pg/ml)	−0.1 ± 3.0	0.0 ± 1.5	0.47
GFR (ml/min)	3.9 ± 24.3	−5.3 ± 26.1	0.14
Renal plasma flow (ml/min)	13.4 ± 104.0	−30.7 ± 121.8	0.23
Urine flow (ml/min)	2.1 ± 3.8	1.4 ± 3.8	0.55
Sodium excretion (mEq/min)	97.0 ± 182.5	92.5 ± 185.0	0.90
Urinary cGMP excretion (pmol/min)	173.4 ± 517.9	1851.0 ± 1386.4	<0.01

Values are shown as mean ± SD.

Abbreviations: ANP, atrial natriuretic peptide; BNP, brain natriuretic peptide; BP, blood pressure; cGMP, cyclic guanosine monophosphate; GFR, glomerular filtration rate; LAV, left atrial volume; LVEF, left ventricular ejection fraction; RVSP, right ventricular systolic pressure.

### 3.3 Neurohumoral response to volume load

With tadalafil alone, there was no significant change in ANP (39.7 vs. 42.9 pg/ml, *p* = 0.69) and a trend for increase in plasma cGMP (4.1 vs. 5.8 pmol/ml, *p* = 0.06) in response to VL (Table 2).

Tadalafil plus SC BNP combination, there was a trend for decrease in ANP (43.5 vs. 31.7 pg/ml, *p* = 0.06) and an increase in cGMP (4.4 vs. 15.7 pmol/ml, *p* < 0.01) and BNP (79.9 vs. 603.7 pg/ml, *p* < 0.01) in response to VL (Table 2).

Tadalafil plus SC BNP combination resulted in elevated plasma BNP (523.8 vs. 5.9, *p* < 0.01) (Figure 3b) and a greater cGMP response to VL as compared to tadalafil alone (11.3 vs. 1.7 pmol/ml, *p* < 0.01) (Figure 3c).

### 3.4 Adverse events

No subjects receiving tadalafil and SC placebo had adverse events. With administration of tadalafil and SC BNP, two subjects (10%) experienced hypotension that subsequently resolved with saline infusion, and one subject (5%) had diarrhea that self‐resolved.

## 4 DISCUSSION

This study characterizes the acute cardiac and renal adaptations of PDEVI alone and in combination with SC BNP in response to VL in subjects with PDD. This is the first‐in‐human study to define the effects of such cGMP modulation in PDD. Our goal was to determine if tadalafil alone or in combination with SC BNP would result in improved cardiac and renal adaptations to VL in PDD subjects. Our findings showed that PDEVI alone did not improve cardiac adaptation to VL. However, the combination of PDEVI and exogenous SC BNP resulted in improved cardiac adaptation to VL. With regard to renal response, there was no difference in either group.

Preclinical diastolic dysfunction (PDD), which resides in ACC/AHA Stage B heart failure, and is defined as ejection fraction greater than 50%, with moderate/severe diastolic dysfunction and no diagnosis or signs and symptoms of heart failure, is a common entity that disproportionately affects older patients with cardiovascular comorbidities, and results in increased risk of progression to symptomatic HFpEF (Abhayaratna et al., 2006; Lam et al., 2011; Lauer et al., 1992; McKie et al., 2011; Mureddu et al., 2012; Redfield et al., 2003; Wan et al., 2014; Wang, Evans, et al., 2003; Wang et al., 2003b; Yancy et al., 2017). There are currently no approved therapies for PDD or HFpEF. The PhosphodiesteRasE‐5 Inhibition to Improve CLinical Status and EXercise Capacity in Diastolic Heart Failure (RELAX) trial investigated the PDEV inhibitor sildenafil and showed that there was no clinical benefits in mortality or improvement in exercise capacity in patients with heart failure with preserved ejection fraction (Stage C HF) (Borlaug et al., 2015; Redfield et al., 2013). The current study provides insight as to why there might not have been any clinical benefit in PDEV inhibition alone, as our findings showed no improvement in cardiac adaptation to VL with tadalafil alone, specifically to LV EF, LAV, and RVSP. In contrast, with the combination of PDEVI and BNP administration, there was improved cardiac adaptation to VL, as shown by improvement in ejection fraction and reduction in LAV and RVSP. A borderline significant reduction in ANP (43.5 vs. 31.7 pg/ml, *p* = 0.06) may suggest a decrease in cardiac filling pressures with PDEVI and BNP. In the PDEVI alone group, there was no change in ANP with VL (39.7 vs. 42.9 pg/ml, *p* = 0.69).

The angiotensin receptor neprilysin inhibitor (sacubitril/valsartan) is currently approved for the treatment of heart failure with reduced ejection fraction (HFrEF) (Yancy et al., 2017). The PARAMOUNT trial found that sacubitril plus valsartan compared to valsartan alone resulted in greater reduction in NT‐pro BNP levels (Solomon et al., 2012). However, in the larger PARAGON trial, when sacubitril valsartan was compared to valsartan alone, there was no improvement in rate of hospitalization or mortality among HFpEF patients with an ejection fraction of 45% or greater (Solomon et al., 2019).

These findings may be attributed to the fact that endogenous NP levels are lower in HFpEF compared to HFrEF (Maisel et al., 2003; Santhanakrishnan et al., 2012; Veldhuisen et al., 2013). Therefore in HFpEF, neprilysin inhibition alone is less effective since endogenous ANP and BNP are lower. In addition, prior studies show that exogenous natriuretic peptides such as ANP are physiologically less effective in vasodilation in HF compared to normal subjects (Hirooka et al., 1990). We hypothesize in HFpEF, exogenous administration of active peptide BNP is needed, and our study results showed that addition of SC BNP to PDEVI improves cardiac adaptation to VL. These findings are also consistent with prior data that shows chronic BNP administration results in improved diastolic function and renal function with VL (Wan et al., 2016).

We have previously reported that there is impaired cardiorenal response to VL in patients with PDD as compared to normal subjects (McKie et al., 2011). Renal cGMP activation was decreased with reduced urinary sodium excretion in subjects with PDD when exposed to VL.

Given that this was a small proof of concept randomized double blinded placebo controlled cross over study with 20 subjects, and the focus is on investigating the effects of PDEV inhibition, either alone or added to BNP, we wished to maximize enrollment in groups receiving PDEV inhibition and thus did not have a BNP alone study arm. Prior studies have demonstrated that chronic administration of BNP alone in PDD results in significant activation of cGMP, natriuresis, and improvement in diastolic function (McKie et al., 2011; Wan et al., 2016). The degree of increase in plasma cGMP with volume expansion at the 10 µg/kg dose of BNP was 10.4 and 7.3 pmol/ml in the prior studies investigating BNP alone in PDD. This is compared to an increase of 15.7 pmol/ml with VL in PDD in this study when combination Tadalafil plus BNP was administered. While these values cannot be directly compared due to variability in study protocol, these findings suggest that combination PDEV inhibition with BNP does have a significant effect on neurohormonal findings in PDD.

While there was no cardiac function improvement in response to acute VL with tadalafil alone, there was a significant improvement in cardiac function with combination tadalafil and BNP administration in subjects with PDD. However, there was no change in renal function which may be due to the decrease in blood pressure observed with tadalafil alone or in combination with BNP. This results in reduced renal perfusion pressure and distal sodium delivery, with worse sodium excretion (Haas et al., 1986). When the renal vasodilatory and vasoconstrictive mechanisms are dysfunctional, GFR may be reduced (Shipley RE & Study RS, 1951).

There are several potential mechanisms for the benefits in tadalafil, as a regulator of nitric oxide levels, when combined with exogenous BNP administration. PDEV hydrolyzes cGMP coupled to nitric oxide, but not cGMP associated with natriuretic peptides in cardiomyocytes (Lee et al., 2015). However, PDEV may mediate not only nitric oxide levels, but also exert an important role in cGMP degradation through the natriuretic peptide pathway in the aorta smooth muscle cells (Zhang et al., 2019). Furthermore, PDEV may have differential effect in certain organ systems rather than a systemic effect. There is pulmonary vasodilation mediated by cGMP with PDEV, but in the kidneys there is independent BNP responses not potentiated by PDEV (Baliga et al., 2008; Frees et al., 2021). All of these studies suggest that there is a complex action of PDEV that may include both the nitric oxide and natriuretic peptide‐dependent cGMP pathways with differential effects based on organ system.

In summary, the results demonstrated that in human subjects with PDD, tadalafil and BNP combination compared to tadalafil alone resulted in cardiac function improvement, but no change in renal function with acute VL.

### 4.1 Study limitations

A limitation of this study is that only one dose of tadalafil and SC BNP was tested. Future studies are needed to further evaluate the effects of lower, non‐hypotensive doses of tadalafil and SC BNP. This is also a study assessing acute physiological derangements and hence, may not reflect the long‐term effects of tadalafil and SC BNP on cardiorenal function and neurohormonal effects in PDD. This study was limited by a shorter duration of investigation. Additionally, PDD is heterogenous, and future studies require selection of more specific patient groups from this diverse population.

## 5 CONCLUSION

In human subjects with PDD, PDEV inhibition alone with tadalafil resulted in no improvement in cardiac adaptation to VL. However, the combination of PDEV inhibition and exogenous SC BNP resulted in improved cardiac adaptation to VL.

## DISCLOSURES

Dr. Chen and Dr. Burnett have patented and licensed designer natriuretic peptides.

## CONFLICT OF INTEREST

The authors have no conflict of interest to report.

## AUTHOR CONTRIBUTIONS

All authors have read and approved the manuscript, and have contributed to the design, collection of data, analyses, writing, and/or review of the manuscript.
